# The Impact of Scleral Lenses on Intraocular Pressure

**DOI:** 10.3390/jcm15041635

**Published:** 2026-02-21

**Authors:** Langis Michaud

**Affiliations:** École D’optométrie, Université de Montréal, Montreal, QC H3T 1P1, Canada; langis.michaud@umontreal.ca

**Keywords:** scleral lenses, intraocular pressure, pneumotonometry, episcleral veins, Bruch’s Membrane Opening-Minimum Rim Width (BMO-MRW)

## Abstract

**Background:** In 2016, Charles McMonnies advanced a theory positing that the use of scleral lenses might result in an elevation of intraocular pressure (IOP) due to the compression of the episcleral veins, consequently diminishing the eye’s capacity for draining aqueous humor. Alternative drainage pathways are capable of compensating only for 10–30% of the aqueous humor that requires drainage. Then it remains a quantity of fluid trapped in the anterior chamber. Recent data has demonstrated that the scleral lenses wear results indeed in an augmentation of the anterior chamber volume and a reduction of the iridocorneal angle, concomitant with a compression of Schlemm’s canal. Assuming that aqueous humor production remains constant, this imbalance between inflow and outflow can only lead to an increase in intraocular pressure. **Methods:** Several studies have attempted to answer this question over the past 10 years. Most authors have encountered the inherent difficulty of measuring IOP while the lens is still in place. Others were performed without waiting for the required time (>4 h of wear) for the lens to exert its maximum compression, thus minimizing their impact. Some attempted to assess IOP via the sclera (pneumotonometry), a technique known to give variable results and hard to reproduce. Ultimately, there are few reliable ways to assess IOP. One of them is by directly observing changes in the optic nerve structure over time. **Results:** These works indicate that there is indeed a moderate increase (<5 mmHg) in IOP. Could this be causing neuropathy and long-term negative impacts for patients who may be at risk? Based on the clinical experience of those involved in the field for many years, it is unlikely that IOP variations may have an impact on a healthy optic nerve. However, glaucoma patients or those at risk could be adversely affected in the long term. **Conclusions:** It is still too early to determine, without a doubt, the actual impact of the likely increase in IOP resulting from the structural changes caused by wearing scleral lenses Further work is therefore urgently needed to document these longitudinal changes.

## 1. Introduction

In 2016, Charles McMonnies raised a question that has remained unanswered ever since: does wearing scleral lenses affect IOP [1]? In raising this question, McMonnies suggests that the pressure exerted by the lens on the episcleral veins may reduce the drainage of aqueous humor, contributing to a temporary increase in IOP during lens wear. Research has therefore been undertaken, but the great difficulty remains in accurately assessing IOP while the lens is still in place. After nearly 10 years of investigation, the conclusions of this research remain mixed, and it is difficult to reach a consensus on the data collected.

This article aims to take stock of the current situation and attempts to translate the evidence on which practitioners should rely into clinical terms.

## 2. Methodology

This article is based on a review of all articles published since Charles McMonnies’ article in 2016. The search was conducted using databases (PubMed, Medline, Web of Science) with the keywords scleral lens or scleral contact lens in combination with intraocular pressure, tonometry, pneumotonometry, trans palpebral tonometry, or optic nerve.

A total of 931 articles were identified, including 268 duplicates or triplicates. Of the remaining 663, the abstracts were read to determine whether they were relevant to the present study based on the following inclusion criteria: use of scleral lenses and measurement of intraocular pressure using various methods before, during, or after wearing these lenses in any clinical population. Articles that did not include direct or indirect pressure assessment were excluded. At the end, 24 articles were kept for analysis.

## 3. Mechanisms of Intraocular Pressure (IOP) Variation

Before even considering the impact of scleral lenses on IOP, it is important to review the mechanisms of aqueous humor production and its drainage pathways to fully understand the factors involved that could contribute to changes in IOP while wearing scleral lenses.

IOP is primarily determined by the dynamic equilibrium between the rate of aqueous humor formation by the ciliary epithelium and its resistance to outflow through conventional and unconventional pathways [2]. Aqueous humor is produced in the ciliary processes by a combination of passive diffusion and ultrafiltration of plasma across fenestrated capillaries into the ciliary stroma, followed by active trans-epithelial secretion across the pigmented and non-pigmented ciliary epithelium, driven largely by ionic transporters and carbonic anhydrase–dependent bicarbonate movement, which establish osmotic gradients that draw water into the posterior chamber [3]. The fluid then flows from the posterior chamber through the pupil into the anterior chamber, where convective currents created by cornea–iris temperature gradients facilitate mixing and distribution of nutrients to avascular tissues [4], while the constant production rate of approximately 2–3 µL/min in the healthy adult eye provides a steady inflow component that contributes to a physiological IOP around the mid-teens in mmHg [5].

Outflow of aqueous humor occurs via two principal routes whose resistive properties critically regulate IOP. The conventional, pressure-dependent pathway accounts for the majority of outflow and involves passage of aqueous humor from the anterior chamber through the trabecular meshwork at the iridocorneal angle, into Schlemm’s canal, then through collector channels and episcleral veins back to the systemic circulation. Alterations in trabecular and juxtacanalicular extracellular matrix composition are key determinants of outflow resistance and IOP elevation in glaucoma [6]. The unconventional, largely pressure-independent uveoscleral pathway allows aqueous humor to percolate through the uveal meshwork and ciliary muscle, traverse the suprachoroidal space, and exit through the sclera, and its relative contribution to total outflow decreases with age but can be pharmacologically enhanced (for example by prostaglandin analogs), thereby lowering IOP by increasing this low-resistance egress route [7].

It has been established that the obstruction of episcleral veins results in an elevated intraocular pressure (IOP) through the reduction of conventional drainage of aqueous humor (AH). AH accounts for approximately 70–90% of the total flow via the trabecular meshwork to these veins. The uveoscleral pathway has been shown to increase its flow to a certain extent, but not fully, as its normal contribution is limited to 10–30% and varies with age or under the effect of drugs [8]. In the absence of complete compensation, this can result in secondary glaucoma, which can damage the optic nerve [9].

## 4. Scleral Lens Impact on Ocular Tissues

Scleral lenses are large, rigid lenses that rest on the conjunctiva, supported by the sclera. When placed on the eye, the lens exerts a pressure on the tissue that is more or less uniform, depending on how well the lens fits and corresponds to the irregular profile of the conjunctiva. In the case of lenses with spherical peripheries, this means that the pressure will be exerted more on the most elevated areas of the conjunctiva, while the deeper areas will be exempt, favoring more tear exchange under the lens. If the peripheries are well aligned in each of the quadrants around the cornea, the pressure becomes more uniform over the ocular surface. This represents the modern way to fit scleral lenses as quadrant specific lenses are preferred by patients for their improved comfort [10]. This type of fitting also helps to reduce build-up debris in the reservoir (mid-day fogging) and this is another reason whytoric peripheral curves are widely adopted. 

Subsequent to application, the scleral lens will become embedded within the conjunctival soft tissue. Following this process, the lens will reach a state of stability within the range of 6 to 8 h post-application [11], a duration which is contingent upon the unique characteristics of both the conjunctiva and the lens. A recent study has shown that the lens sinks approximately 55 µm into the conjunctiva during this whole process [12].

Episcleral vessels are located at a depth of approximately 30–35 µm beneath the conjunctiva. As the lens settles, causing a 55-µm drop in depth, it is logical to deduce that the pressure exerted by the lenses may have an impact on the episcleral veins. As demonstrated, the suction pressure induced by a scleral lens can reach 5 to 18 mmHg [13]. The pressure of episcleral veins has been shown to be 7–11 mmHg. It is also known that when the external pressure equals the intravenous pressure, there is reflux and restriction of outflow [14]. The restriction of the aqueous humor is the consequence of the pressure exerted, which may result in an increase in the IOP.

It should also be emphasized that wearing scleral lenses is accompanied by an increase in the anterior chamber area, which occurs once the lens has stabilized [12]. Cornea becomes steeper under a close area limited by the back surface of the lens. The conditions are then optimal to modify the inflow-outflow balance and consequently the IOP. The outflow may also be penalised, as it was shown that the angle opening distance at 500 μm (AOD500) and the trabecular-iris space area at 500 μm (TISA500) significantly decreased after wearing scleral lenses for 4 h [15]. Schlemm’s canal was also found reduced after several hours of lens compression [16].

Episcleral venous pressure (EVP) is a key determinant of the IOP. Alteration or compression of the episcleral vasculature can change both the pressure inside of the episcleral vessels and then consequently the IOP [17].

The diameter of a vast majority of the scleral lenses plays little role in this mechanism, since they are designed using curves and all have a landing contact at a chord between 13.5 and 14.5 mm, near the limbus. This point of first contact between the lens and the ocular surface defines the primary functional diameter of the lens (PFDL). More specifically, the PFDL is the diameter of a scleral lens defined by a chord of where the lens first contacts or “lands” on the ocular surface along the same meridian [18]. The entire weight of the lens creates pressure in a limited area of the conjunctiva, increasing its impact on the underlying tissue.

The few scleral lenses designed with tangential profiles instead of curves are distinct in this regard. In such cases, the larger diameter allows for an increased area of conjunctival support, thereby spreading the pressure on the tissue. The presence of fenestration and/or tear exchange channels in the lens may also contribute to limiting the impact of the scleral lens pressure on the episcleral veins.

## 5. Clinical Findings

Since 2016, 24 articles have been published reporting research findings on the effects of scleral lenses on IOP (see complete list in Appendix A, Table A1). Of these, 12 found no difference in pressure when wearing scleral lenses, while 8 found an increase, and 4 reported mixed results depending on the measuring instruments used. Therefore, the majority of studies appear to show no significant effect on IOP. However, the methodologies and instruments used must be analyzed.

Several studies compare IOP before lens application and after a limited number of hours of wear. The pressure is therefore measured before the lens is applied and after it is removed. However, it appears that outflow restriction only occurs when the lens is worn and circulation in the episcleral veins is immediately restored 10–30 s upon removal [19]. It is then expected to find normal, unchanged IOP at lens removal. Otherwise, this would indicate permanent damage to the vascular network. Although practical, this method (pre-post lens wear) may be questioned as a valid one to assess the impact of lens induced pressure on IOP on the long term. In fact, these studies do not actually measure pressure variation, since pressure returns to normal before the post-wear measurement is taken. As the methods used do not seem appropriate for answering the question of the influence of scleral lenses on IOP, it was decided not to consider all studies based on this inappropriate methodology, leaving 8/24 studies to be discussed. Other authors might take a different stance and consider them, despite their limitations. This could be the subject of further debate.

Several instruments have been used to measure IOP, either directly on the cornea, as is normally the case, or using transpalpebral measurement techniques. Among the latter, pneumotonometry is used but its repeatability may be discussed [20]. Diaton (Diaton; DevelpAll Inc., Staten Island, NY, USA) stands out in particular, as it is known for its high variability and low reliability in its measurements [21]. In general, the scientific community does not consider transpalpebral tonometry to be robust enough for research, although it may have other clinical uses in other circumstances. If the studies relying on Diaton are discarded, it remains 4 studies to analyze.

The final element to consider is the time elapsed before the assessment of ocular pressure. As previously stated, the lenses become stable within a period of 4–8 h of wear. This suggests that prior to this time, the lens-induced compression has not yet been fully applied to the conjunctiva, resulting in an incomplete impact on the episcleral veins and IOP. All the studies remained as the IOP assessment was made after 4h00 of lens wear. No longitudinal studies have been conducted to determine the effects of a potential modest but chronic increase in IOP on eye health. Such studies must be conducted to determine the actual effect of the compression phenomenon associated with wearing scleral lenses. Minor changes, such as an increase in papillary excavation from 0.2 to 0.4 over 5–10 years, may not be detectable during a single examination, but could be significant in terms of neuropathy secondary to wearing scleral lenses.

Among the four studies remaining, three addressed the variation of Bruch’s membrane opening (BMO) at the optic nerve level, as this structure was found to be correlated with variations in intraocular pressure (IOP) [22]. One study did identify a small increase in IOP, but it was not statistically significant [23]. Two other studies found that IOP increased based on the variation in Bruch’s membrane opening minimum rim width (BMO-MRW). Both a normal population [24] and a population diagnosed with keratoconus [25] were evaluated, and the conclusion was the same: there is a significant variation in BMO-MRW during scleral lens wear, once the diurnal variation has been taken into account. The main issue here is to translate this variation into a value of IOP in mmHg as there is no consensus. The authors based their findings on studies conducted in both humans and animals and determined that a ratio of 0.6 mmHg could be used considering this variability. This results in an estimated increase in IOP of at least 4 mmHg. when wearing scleral lenses. This is obviously an estimate, and other methods of correlation with other structures of the optic nerve could lead to higher or lower values. Although imperfect, this estimate seems to be logical and takes into account current scientific knowledge, with all the limitations that this may entail.

At least, the studies selected have the merit of having directly observed the impact of wearing scleral lenses on the optic nerve. Any variation in IOP can have adverse effects on this structure. It is therefore relevant to pay close attention to this when assessing the impact of wearing scleral lenses on the eye.

It is obvious that other authors may, in future, question this ratio and therefore the conclusions that should be drawn from the cited studies. It is possible that new technologies may enable better in situ assessment of IOP variation during scleral lens wear. Such advances are not only expected, they are necessary to improve our understanding of the long-term impact of scleral lenses on the optic nerve.

Approaching this attempt to achieve what currently appears impossible, Cheung et al. [26] demonstrated that IOP increased rapidly when the scleral lens was fitted, using a fenestrated lens. The latter only exerts pressure on the episcleral veins, without any suction effect from the reservoir on the cornea. This result seems to reinforce McMonnies’ hypothesis.

The last study to consider tried another approach, measured transconjunctival pressure using a Schiotz tonometer. This is not the most accurate method, but it is a recognized way of measuring IOP, although the subject must be lying down horizontally, which can influence the fluids in the eye. Habitually IOP is found increased under these positions [27].

### Clinical Consequences

It therefore appears that wearing scleral lenses may be associated with a modest but definite increase in IOP, when measurements are made after full lens stabilization, with the lens in situ and results compensated for diurnal variation.

In general, there is considerable variability between individuals, depending on the lenses worn, the lenght of wear, their anatomical structure, etc. IOP increase found in studies analyzed takes into account the physiologically normal diurnal variation found in both younger individuals [28] than the older ones [29]. This variation and balance in IOP becomes less effective in glaucoma patients, although they also experience higher pressure at night.

A healthy eye and a robust optic nerve will undoubtedly be able to cope with an increase of less than 5 mm in IOP during the day. It would be surprising to cause neuropathy by subjecting the eye to a pressure of 19 instead of 14 mmHg, even if this increase is chronic and persists throughout the entire period of scleral lens wear.

The case of glaucoma patients is different. The last thing that should happen in glaucoma patients is a significant fluctuation in their IOP. Even a slight variation can have significant consequences on the progression of the disease and will obviously require a change in the treatment applied [30].

While it is clear that wearing scleral lenses will not cause de novo glaucoma, it is also clear that glaucoma patients may see an increase in the risk of disease progression when wearing scleral lenses.

Caution must be exercised here, and the risk/benefit ratio must always be considered in the patient’s best interest. If the risk to wear scleral lenses outweighs the expected benefit, other lens options for vision correction or treatment of the patient’s condition should be considered. Conversely, if the benefit of sclerals, as the only option for this patient, outweighs the risk, caution should still be exercised by frequently reviewing the glaucoma patient to ensure that both structurally (appearance of the optic nerve, scans) and functional (visual field) levels, and that their condition does not lead to losses that could have been avoided by fitting other types of lenses.

In all cases, photographic documentation of the optic nerve and careful observation of structural changes over time should be performed in all scleral lens wearers. Changes in excavation or variation in the visual field may be subtle from one year to the next and difficult to quantify without documented comparisons on file.

## 6. Conclusions

Wearing scleral lenses compresses the episcleral network which is likely inducing outflow restriction regardless of the lens size. At the same time, the volume of the anterior chamber increases, and the iridocorneal angles, as well as Schlemm’s canal, are reduced. The combination of these two factors can sum up, although there is considerable variation between individuals. Fitting lenses “loose”, considering adding tear exchange channels or fenestrations constitute good alternatives to alleviate potential negative consequences.

Patients fitted with scleral lenses must be closely monitored, particularly if they have confirmed glaucoma or risk factors for glaucoma (heredity, narrow angle, etc.). That said, scleral lenses remain hopefully a very safe alternative for the vast majority of patients.

## Data Availability

No new data were created or analyzed in this study. Data sharing is not applicable to this article.

## Appendix A

**Table A1:** Summary of Studies Looking at IOP and Scleral Lens Wear.

First Author, Year Published	Diameter of the Lens (mm)	Instrument	Moment/Lens Wear	Findings
**NO IMPACT**				
Vincent 2017 [31]	16.5	ORA NCT		Literature review. Suggests that there may be a slight increase in IOP during wear, which does not alter a healthy eye. Calls for further studies vs. designs and higher-risk populations.
Porcar 2019 [32]	12.6–13.5	ORA	R	No significant difference in ocular biomechanics or IOP 1 year after wearing scleral lenses
Walker 2020 [23]	15.4	S/OCT2 DT RT	W	Variation in IOP and optic nerve appearance but not statistically significant—normal population
Fedotova 2021 [33]	14.9	Monocular RT	R 6h00	No effect of scleral on IOP on removal—normal population
Obinwanne 2020 [34]	16.0	Sch	W 4h00	No significant difference in IOP and AC angle
Kramer 2020 [35]	16.0 to 17.5	GAT	R	No increase in IOP at 1 and 6 months of scleral wear.
Shahnazi 2020 [36]	17.0 to 18.0	TP	R	Corneal thickness increases with lens wear, but no significant change in IOP measured on removal
Nau, Schornack 2022 [10]	18.0	PMT	B, R 2h00	No effect of lens wear on IOP—normal population Spherical vs. toric haptics
Kumar, Shetty 2022 [37]	15.6 to 16.4	NCT-C	R 8h00	No significant variation in IOP on removal. Post-graft population
Macedo-de-Araújo et al. 2023 [38]	16.4	NCT-C	B, R	No significant difference between IOP before and after removal on regular and irregular corneas.
Yang et al. 2025 [39]	15.6	NCT RT	R	No increase from baseline after several months of wear. Participants removed lenses the day before measurement
Queiruga-Piñeiro et al. 2025 [40]	15.8 and 16.8	NCT-C	B, R 2h00	No change in IOP after lens wear
**IOP INCREASED**				
Montalt 2018 [41]	12.6–13.5	ORA	R	Slight increase in IOP (0.2 mmHg) after 1 year following scleral wear.
Michaud 2019 [42]	15.8 to 18	DT	W	Average increase of 5 mmHg during lens wear, unaffected by lens diameter. Wide inter-subject variation.
Aitsebaomo 2019 [43]	15.8	RT	R	Significant increase of 5.8 mmHg after wearing
Cheung 2020 [26]	16.5	RT	W At removal	Difference of 3.5 mmHg when wearing a fenestrated lens, enabling measurement directly on the cornea.
Samaha 2021 [24]	16.0	S/OCT	W 6h00	BMO-MRW is reduced during wearing, Suggests an IOP increase of 5 mmHg- normal population
Formisano 2021 [44]	16.8	GAT DT	B, R, W 8h00	A small but statistically significant IOP increase during lens wear- Keratoconus population
Queiruga-Pineiro 2023 [16]	15.8 to 16.8	MK2 (PAT) DT	R 0–2h00	Slight increase in IOP with scleral lenses but does not seem to be clinically significant in healthy subjects.
Michaud et al. 2025 [25]	16.0–16.5	S/OCT	W 6h00	BMO-MRW became significantly thinner. This related to an increase in IOP up to 5 mmHg. Keratoconus population
**MIXED RESULTS**				
Nau 2016 [45]	15.0	PMT	R 1–2h00	In new wearers, central (cornea) and peripheral (sclera) IOP did not vary significantly, on average, However, there is high variability. Some individuals have IOP > 20 mmHg after wear
Fogt 2020 [46]	15.2 and 18.0	PMT DT	W 1h00	No difference in IOP with pneumotonometry but significant difference with transpalpebral- normal population Measurement technique influences results
Ganjei et al. 2021 [20]	>18.0	TP on sclera, PMT	W	Handheld tonometry and pneumatonometry/KC patients These methods are not repeatable and not reliable
Litvin 2023 [47]	15.6 to 18.0	PMT	B, R 5h00/ 2.5 h	No significant variation in corneal IOP before and after wear or with different lens diameters (5 h). Significant increase in scleral IOP after 2.5 h of wear.

INSTRUMENTS: S/OCT 2—Spectralis OCT 2 (Heidelberg Engineering, Franklin, MA, USA); DT: Diaton (Diaton; DevelpAll Inc., Staten Island, NY, USA); RT: Rebound tonometry (Icare Finland Oy, Vantaa, Finland); Sch: Schiotz tonometer (Wingers, Jungingen, Germany); GAT: Goldman tonometer (Haag-Streit, Germany); TP: Tono-Pen AVIA (Reichert, Depew, NY, USA); NCT-C: Corvis (Oculus, Germany)—biomechanical analyzer; MK2(PAT) or PK: Perkins—(Haag-Streig holding, Harlow, UK); PMT: Pneumotonometry (Model 30 Classic, Reichert Ophthalmic Instruments, Depex, NY, USA); ORA: Ocular Response Analyzer (Reichert, Inc., Buffalo, NY, USA); NCT: Non contact tonometer- (TX-20P, Canon, Amstelveen, The Netherlands, Pays-Bas); MOMENTS: B: before lens wear, W: during lens wear, R: upon lens removal.
